# Recent communication disorders research through the lens of the sustainable development goals

**DOI:** 10.4102/sajcd.v71i1.1079

**Published:** 2024-11-07

**Authors:** Anita Edwards

**Affiliations:** 1 South African Speech Language and Hearing Association (SASLHA), Durban, South Africa

The articles published in the *South African Journal of Communication Disorders* (SAJCD) since January 2024 address various aspects of health, communication, and social support, which are closely aligned with several Sustainable Development Goals (SDGs).

## Health and well-being (Sustainable Development Goal 3)

Several articles focus on health-related issues, particularly concerning hearing and communication disorders.

### Early hearing detection and intervention

An opinion article calls for linguistically and culturally congruent family-centred early hearing detection programmes and emphasises the importance of timely interventions to improve health outcomes for children. This aligns directly with SDG 3, which aims to ensure healthy lives and promote well-being for all at all ages.

### Noise-induced hearing loss

Research on attitudes towards hearing protection in coal mines highlights occupational health risks and the need for preventive measures, contributing to the goal of reducing morbidity from environmental hazards.

## Quality education (Sustainable Development Goal 4)

The articles also touch on educational aspects related to communication disorders such as in the articles, which provide insights about tube feeding in advanced dementia and feeding decisions for children with cerebral palsy. These articles underscore the necessity of training healthcare professionals and align with SDG 4, which promotes inclusive and equitable quality education and lifelong learning opportunities.

## Reduced inequalities (Sustainable Development Goal 10)

A number of articles address disparities in access to healthcare services. The exploration of barriers faced by caregivers in peri-urban communities points to inequalities in accessing essential audiology services. Addressing these barriers is crucial for promoting equal access to health services, as highlighted in SDG 10.

## Partnerships for the goals (Sustainable Development Goal 17)

Collaboration among stakeholders is a recurring theme:

### Stakeholder-informed adaptation guidelines

The article on Enhanced Milieu Teaching emphasises the need for collaboration among educators, therapists, and families to adapt teaching methods effectively. This reflects SDG 17’s focus on partnerships to achieve sustainable development.

## Conclusion

The content of the articles published in SAJCD since January 2024 demonstrates a strong commitment to addressing key issues related to health, education, and social equity. By focusing on early detection and intervention, training healthcare professionals, and addressing access disparities, these articles contribute significantly to advancing multiple SDGs, particularly those related to health and well-being, quality education, reduced inequalities, and partnerships. This alignment underscores the critical role that communication disorders research plays in supporting broader sustainable development efforts in South Africa.

